# Effect of Residual Stress on S–N Curves and Fracture Morphology of Ti6Al4V Titanium Alloy after Laser Shock Peening without Protective Coating

**DOI:** 10.3390/ma12223799

**Published:** 2019-11-19

**Authors:** Xinlei Pan, Xiang Li, Liucheng Zhou, Xiaotai Feng, Sihai Luo, Weifeng He

**Affiliations:** 1 Science and Technology on Plasma Dynamics Laboratory, Air Force Engineering University, Xi’an 710038, China; 2 The First Training Brigade of Shi Jiazhuang Flying College of PLA Airforce, Shijiazhuang 050000, China; 3 Institute of Aeronautics Engine, School of Mechanical Engineering, Xi’an Jiaotong University, Xi’an 710049, China

**Keywords:** crack initiation, mean stress, fatigue performance, laser shock peening, Ti6Al4V titanium alloy

## Abstract

In this paper, the effect of residual stress on the stress–life (S–N) curve and fracture morphology characteristics of Ti6Al4V titanium alloy after laser shock peening (LSP) without protective coating was experimentally investigated. The fatigue test and residual stress measurement were conducted on specimens before and after the LSP process. It was shown that LSP produced a high-amplitude compressive residual stress field on the surface of the specimen. After the LSP process, the fatigue life limit was increased by 16%, and the S–N curve shifted upward. Then, based on the theory of mean stress, the mechanism whereby the compressive residual stress improves the fatigue life of Ti6Al4V titanium alloy was analyzed. It indicated the improvement in fatigue life was because of the high-amplitude compressive residual stress on the surface and in depth induced by LSP to reduce the tensile stress produced by external loading. In addition, the scanning electron microscope (SEM) pattern of fatigue fracture demonstrated distinct differences in the fracture morphology before and after LSP. After LSP, the crack initiation sites of the samples moved to the subsurface where it was difficult for fatigue cracks initiating here. Moreover, after the LSP process, there were high density of fatigue striations and many secondary cracks on the fracture of the treated specimen.

## 1. Introduction

With advantages like high strength, excellent corrosion resistance, and good compatibility with composites, titanium alloys have wide application potentiality in the field of the aero-astronautical industry [1,2]. However, a serious weakness with titanium alloys is that owing to their sensitivity to stress concentration, fatigue fractures often occur in service [3]. To improve the stress field condition and prolong the fatigue life of titanium alloys, many surface strength technologies are put forward, such as shot peening (SP) [4], deep rolling (DR) [5], and laser shock peening (LSP) [6,7,8]. Of these, LSP technology has become a hot topic in the field of surface modification. After the process, high-amplitude compressive residual stress is introduced into the materials, which results in the substantial improvement of fatigue life of the metallic components [9]. It has been concluded by many researchers that higher amplitude and deeper residual stress distribution has a great effect on the increment of the fatigue life of metallic components by delaying and preventing crack initiation and propagation [7,8,10,11,12]. In recent years, numerous studies about LSP were carried out. The results showed that the LSP process could be applied in a variety of metallic materials, and the specimens with LSP treatment always exhibited effectively improvement of material fatigue life [13,14,15,16,17,18]. Gao and Luong [19,20] investigated the high-cycle fatigue performance of 7075-T7451 aluminum alloy with LSP and SP treatment. The results indicated that the deeper compressive residual stress (CRS) layer produced by LSP was more effective to improve the fatigue life of the alloy. Zhang et al. [6] investigated the fatigue life of Ti6Al4V titanium alloy peened by different times of laser shocks. Their results showed greatly enhanced fatigue strength with the increasing number of laser shocks, which was attributable to the elevated compressive residual stress after increasing the shock number. Zabeen et al.’s [21] investigation about the fatigue crack initiation of LSP-ed Ti6Al4V titanium alloy found that high-amplitude compressive residual stress was generated after LSP, which could lower the stress concentration at the zone damaged by foreign objects and inhibit the initiation and propagation of cracks. In addition, rotating bending fatigue experiments conducted by Shiozawa [22] showed that the limit of stress amplitude for S-mode failure significantly increased owing to the compressive residual stresses produced by LSP. We can see from the above investigations that the LSP process can produce higher-amplitude compressive residual stress into the materials, which is very beneficial in extending the fatigue life of metallic components.

However, as an advanced, competitive surface treatment, the efficiency of LSP is restricted by a negative point: During the LSP process, the protective coating suffers from the laser ablation and is damaged severely. It needs frequent replacement. Moreover, after the LSP, the removal of that layer is usually a time-consuming activity, which leads to increased production costs in industrial applications. To solve this problem, laser shock peening without coating (also known as LSPwC) has received more attention. However, there is still limited literatures on the LSP process. Therefore, in order to promote the widespread application of the LSP process and give instructions around the technical optimization of LSP without a coating process, there is a need to investigate the CRS distribution induced by LSP without coating and its effects on stress–life (S–N) curves and the fracture morphology of Ti6Al4V titanium alloy.

With this in mind, this study is to systematically investigate the residual stress distribution after LSP and its effects on the S–N curve and fracture morphology characteristics of Ti6Al4V titanium alloy. The mechanism whereby the residual stress induced by LSP improves the fatigue life of Ti6Al4V titanium alloy was analyzed. Firstly, compressive residual stress distribution of the specimens treated by LSP was tested using X-ray diffraction (XRD). Second, a high-cycle fatigue test was performed on Ti6Al4V titanium alloy to obtain stress–life (S–N) curves. Then, based on the theory of mean stress, the role of residual stress in the fatigue behavior of Ti6Al4V titanium alloy was discussed in detail. Finally, the fracture morphology was observed by a scanning electron microscope (SEM).

## 2. Materials and Methods

### 2.1. Materials and Specimens

Ti6Al4V titanium alloy is used in this work, which is an α + β type double-phase medium strength alloy. Its chemical composition is shown in Table 1, basic physical parameters are listed in Table 2, and basic mechanical properties in Table 3 (Data from [23]).

### 2.2. LSP Processing

**Figure 1:**
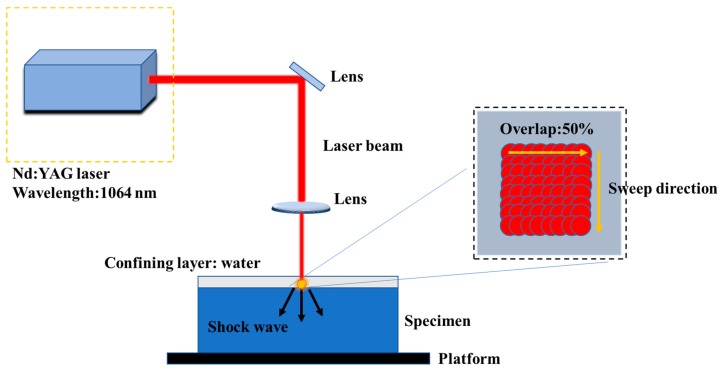
Diagram of laser shock peening (LSP) process.

**Figure 2:**
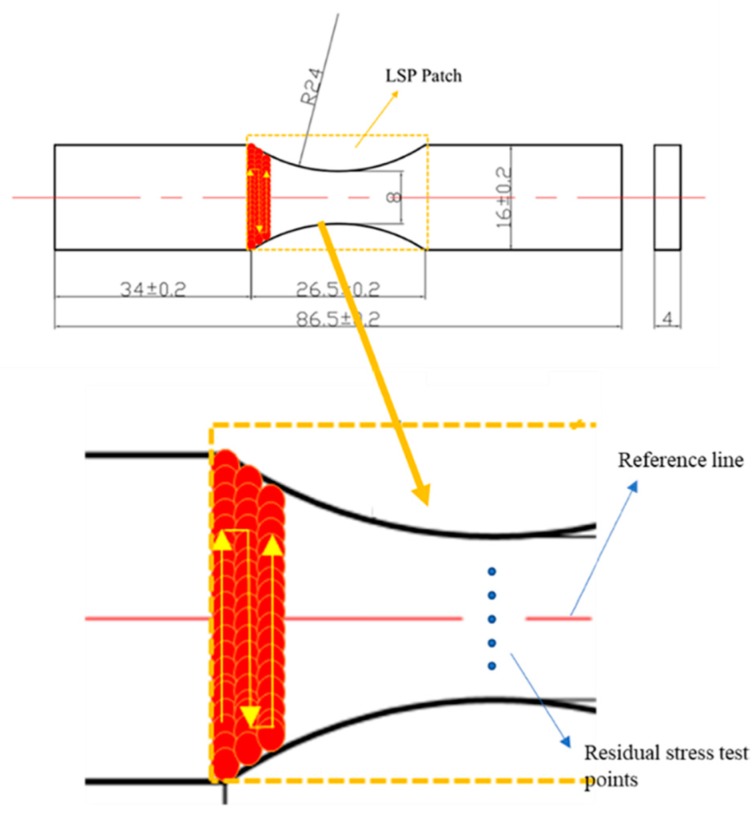
LSP-ed specimen showing the peened patch area.

The schematic diagram of the LSP process is illustrated in Figure 1. The laser used for LSP processing is an Nd:YAG laser named SGR-EXTRA, which is designed by ourselves. Its fluence was 88 J/cm^2^ and power density was 8.85 GW/cm^2^. As can be seen, high-energy shockwave is induced on the material surface by the laser pulse with 1064 nm wavelength and 10 ns pulse width. The overlap ratio was 50%. The laser beam size used was 2.4 mm. The flowing water (thickness 1mm) was taken as the confining layer. During the fatigue test, the dimensions of the using fatigue specimens with the peened patch area are shown in Figure 2. The laser scanning path and overlap during the LSP process are also shown. The two-sided LSP process treatment was carried out on the specimens to prevent the deformation with one-sided LSP process treatment. Based on a large number of previous experiments about LSP parameters and its effects on residual stress distribution, the LSP parameters was determined. In this process parameters, high-amplitude compressive residual stress can be introduced into the material while better surface integrity is also obtained. The final selected LSP parameters are shown in Table 4.

### 2.3. Residual Stress Test

The residual stress was tested using the Proto-LXRD X-ray diffractometer (Ottawa, Canada) with the sin2ψ method. A single axis goniometer with Cu–Kα X-rays was used during the test. Measurement points were selected on the surface with equal distance (1 mm) from the reference line in the LSP regions as shown in Figure 2. To ensure the reliability of the residual stress test of the Ti6Al4V titanium alloy specimen before and after LSP, for each measured point three measurements were conducted and the average was used. Before each measurement, a standard sample was used to test for calibration of the instrument. Table 5 shows the measurement parameters during the test. For the depth stress test, PROTO’s POLISHER 8818 V-3 electropolishing machine (Shanghai, China) was used to remove the surface material layer by layer. Although this method will lead to a residual stress relaxation, the magnitude of the residual stress relaxation is much smaller compared with the compressive residual stresses induced by LSP. It is reasonable to neglect its effect. The composition of the polishing solution was 10% HClO_4_ + 90% CH_3_OH [24].

### 2.4. High-Cycle Fatigue Test and Fractography Observation

To further analyze the fatigue behavior of Ti6Al4V titanium alloy after LSP, a high-cycle fatigue test was conducted. In this paper, the fatigue limit was measured via the up-and-down method, and the S–N curve was obtained via the group method. The details of these two methods would be introduced in the Section 3.2.1 and Section 3.2.2. The high-cycle fatigue test was conducted on a QBG-100 high-frequency fatigue tester (Jilin, China). The testing machine has a frequency of around 110 Hz. The stress ratio R was set as 0.1. According to the loading force and the initial cross-section area of the specimen, the loading fatigue stresses were calculated. In order to assure the reliability of the results, all the tests were carried out at least twice and the average was taken as the final result. At last, a scanning electron microscope (JEOL/JSM-6360LV, Tokyo, Japan) was used to observe the fracture morphology of Ti6Al4V titanium specimens. Acceleration voltage was 20 Kv and the spot size (diaphragm) was 30 um.

## 3. Results and Discussion

### 3.1. Residual Stress Distribution after LSP

**Figure 3:**
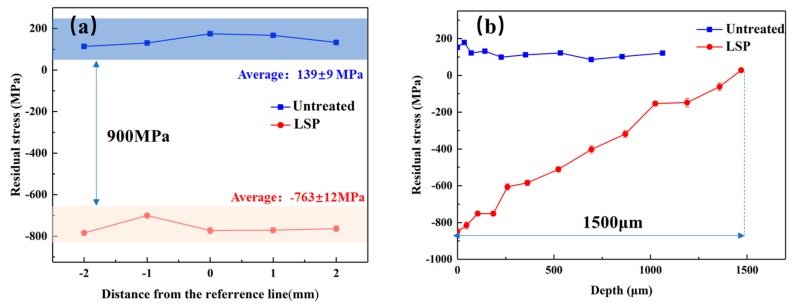
Residual stress distribution of specimens with and without LSP. (**a**) Surface; (**b**) depth.

The residual stress distribution of the material after LSP is shown in Figure 3. Figure 3a shows the surface residual stresses distribution of the untreated and LSP-ed specimens. From it, we can see that there is a tensile residual stress (about 139 ± 9 MPa) appearing on the surface of the untreated specimen. It is analyzed that the tensile residual stress may be introduced during the material machining process such as hot-rolling, cutting, and polishing. However, after LSP, a high-amplitude compressive residual stress field was introduced in the surface layer. The average residual stress was –763 ± 12 MPa. The surface compressive stresses values of specimens with LSP treatment increased by around 900 MPa.

The residual stress distribution in depth is shown in Figure 3b. The residual stress value in the depth is measured by gradual stripping [25], and the average of the three measurements in the same depth is taken as the final compressive residual stress value in this depth. As shown in Figure 3b, the maximum compressive residual stress appears on the material surface. A compressive residual stress layer with an affected depth of 1500 um is induced and it still maintains a high-amplitude compressive residual stress of about −600 MPa at the depth of 200 um. As the depth increases, the value of compressive residual stress reduces gradually. This is because the shock pressure decreases with the increased depth owing to the damping effect of material, and less plastic deformation is generated [12].

### 3.2. Characteristics of the S–N Curve after LSP

#### 3.2.1. Fatigue Limit measured by the Up–Down Method

**Figure 4:**
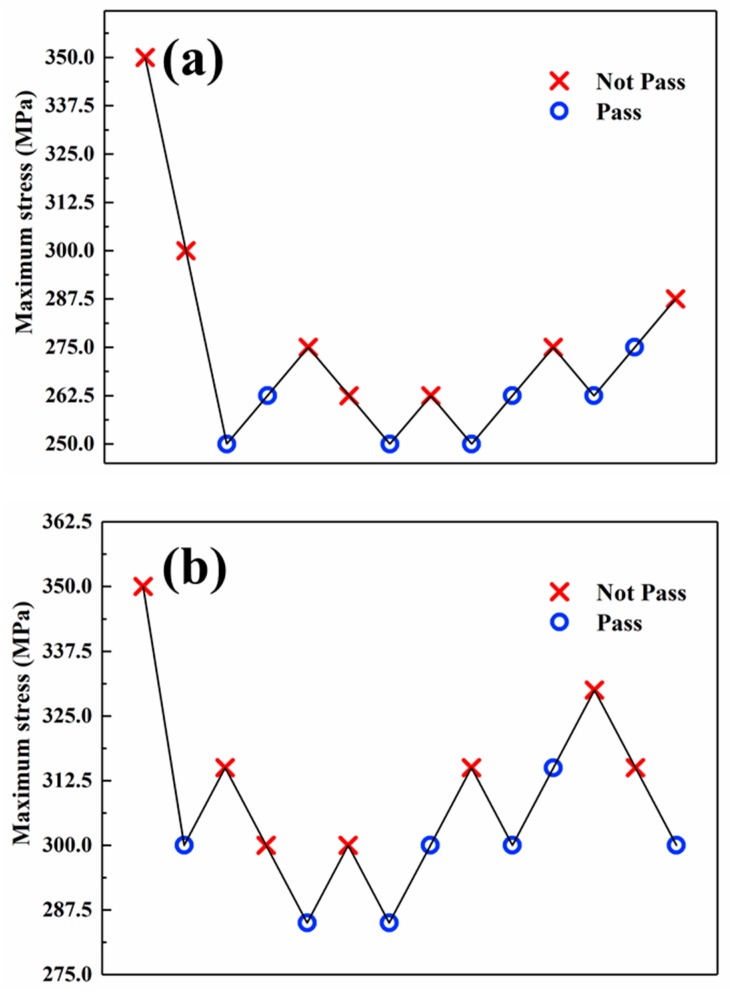
The results of fatigue test of the specimens with and without LSP. (**a**) Untreated; (**b**) LSP-ed.

According to the standard (GB/T 24176—2009) [26] of the up-and-down method of material fatigue, the fatigue test was conducted on the high-frequency fatigue tester. During the fatigue test, a 10^7^ cycles number was considered to be the theoretical critical point of fatigue strength. Generally, the stress level starts at a higher value which is 30–40% of yield limit of material when the reference of fatigue strength is unknown, and then decreases. On the basis of the data in Table 3, the initial stress level was selected as 350 MPa. The next stress level depends on the test result of previous specimen. If the previous specimen fails before 10^7^ cycles number, then the next specimen’s fatigue test should be conducted at a lower stress level. If it is not, the next stress level should be increased. And the stress increment is central for the up-and-down method. The principle that we adopted in the test was as follows: Firstly, an approximate interval of fatigue limit was needed. According our previous research, it is appropriate to set the first stress increment as 50 MPa. When the first pair of opposite results (pass or not) being appeared, the following stress increment would be adjusted as 5% of the previous stress level. For example, the untreated specimens both failed at the stress level of 350 MPa and 300 MPa. But when the stress level decreased to 250 MPa, the specimen was not failed. So the following stress increment would be changed from 50 MPa to 12.5 MPa. This rule also applied the test of the LSP-ed specimens and accordingly its stress increment was adjusted as 15 MPa. From then on, the stress increment would remain unchanged until all the tests were completed. Counting from the first pair of opposite results, we determined 12 samples and divided them into 6 groups. The experimental results are plotted in Figure 4.

During the whole fatigue test, twenty-eight specimens were prepared for the test. Half were treated by LSP and the other half were untreated. As shown in Figure 4, the failed specimens were marked as “×” and the non-failed specimens were marked as “∘”. For the untreated specimens, six different stress levels were used while the LSP-ed specimens was five. These results were satisfied to the standard [26]. There were both seven failed specimens and seven non-failed specimens in the test whether for the untreated specimens or LSP-ed specimens. According to the standard [26], it can be calculated that at 95% confidence level the fatigue limit of untreated specimen is 264 MPa, while the fatigue limit of LSP-ed specimens is 305 MPa. The fatigue limit of Ti6Al4V titanium alloy was improved by 16% after LSP.

#### 3.2.2. S–N Curves Measured by the Group Method

**Figure 5:**
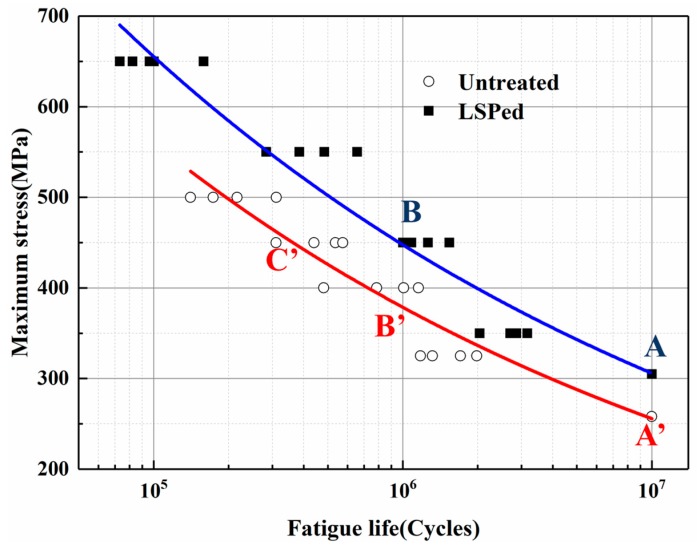
S–N fatigue life curves before and after LSP(R = 0.1).

According to the technology standard (HB/Z112-86) [27], thirty-two fatigue samples were tested for their fatigue life at different stress levels. The fatigue test samples were divided into two groups, one of which was untreated and the other group was treated by LSP. During the test, we set five stress levels. Because the fatigue limit had been tested by the up-and-down method, the results were directly used as the fatigue life at 1 × 10^7^ (A and A’ in Figure 5). In order to ensure that the fatigue results had 95% confidence level, at each fixed stress level four specimens were used in the test. For the sake of obtaining a more complete S–N curve, during the selection of stress level the principle that we took was to try to make the number of cyclic loading scattered at 1 × 10^5^, 5 × 10^5^, 1 × 10^6^ and 5 × 10^6^. The test data at each fixed stress level was processed by calculation of average. Power model of non-linear regression was adopted to fit the curve. Data from the test were plotted in the form of S–N curves. The experimental results were shown in Figure 5.

From the Figure 5, we can clearly see that the two curves both had the same trend. As the maximum stress increased, the fatigue life declined. But there were remarkable differences between LSP-ed and untreated specimens. Under the same maximum stress level condition, the overall S–N curve of specimens treated by LSP moved right compared with untreated specimens. This indicated at the same maximum stress level the average fatigue life of LSP-ed was improved. For example, the specimen without LSP treatment exhibits an average fatigue life of 365,300 cycles at a maximum stress of 450 MPa, which is marked as C’ in Figure 5. However, the specimen with LSP treatment recorded higher performance in fatigue endurance with 1,002,975 cycles at the same maximum stress, which is marked as B. Compared with untreated specimens, the fatigue life of LSP-ed specimen is increased by 235% at the maximum stress of 450 MPa. When it came to the fixed fatigue life condition, the S–N curves move upward after LSP. For example, at a fatigue life of 1 × 10^6^ cycle number the maximum stress of LSP-ed specimen can reach 450 MPa (B point in Figure 5). But for the untreated specimen, the maximum stress is only 380 MPa under the same number of cycles (B’ in Figure 5). The increment of maximum stress after LSP is about 18.4%. For the life of 1 × 10^7^ (A and A’ in Figure 5), the fatigue limit after LSP is improved by about 16%, which is obtained by the up–down method. In conclusion, after LSP process the fatigue life of the LSP-ed specimen is markedly improved.

### 3.3. The Effect of Residual Stress on the S–N Curves

In general, the effects of residual stress on fatigue limit can be evaluated through the viewpoint of mean stress, which is usually investigated according to the Goodman theory [28,29,30]. According to the investigation, the following equation can be obtained:

(1) $$\Delta \sigma_{w}^{r}=-m\sigma_{r},$$

where $\Delta \sigma_{w}^{r}$ is the change of fatigue limit caused by LSP, m is the mean stress sensitivity coefficient which depends on material, and $\sigma_{r}$ is residual stress of the material. As we mentioned above, there is a tensile residual stress (about 139 ± 9 MPa) existing on the surface of the specimen without LSP. From the Equation (1) we can clearly see that the tensile residual stress will result in the decrease of fatigue limit. In addition, the external loading put extra tensile stress on the specimen during the fatigue test, which leads to the further increase in the tensile stress on the specimen. But for the LSP-ed specimen, the severe plastic deformation of the material occurred under the ultra-high pressure shock wave induced by laser and the high amplitude compressive residual stress was introduced into the materials. According to the Equation (1), the fatigue limit will be improved. Besides, due to the existence of compressive residual stress induced by LSP, the tensile stress produced by external loading was decreased, and thus the real stress which the specimen suffered from during the fatigue test was lower than the tensile stress which external loading exerted. Although the compressive residual stress would be relieved partly as the number of fatigue cycles increased, there is always a compressive residual stress layer remained in the material [31]. In addition, compressive residual stress also increases the threshold value of the crack initiation. The crack initiating would be prevented in the compressive residual stress affected layer, which remarkably reduces the occurring probability of fatigue crack initiation [32,33,34,35]. For the microscopic cracks already established in the materials, compressive residual stress can arrest the crack into closure and maybe lead to a non-propagating crack [36,37]. Thus, the high amplitude compressive residual stress induced by LSP is beneficial to improve the fatigue life.

### 3.4. Fracture Feature after LSP

**Figure 6:**
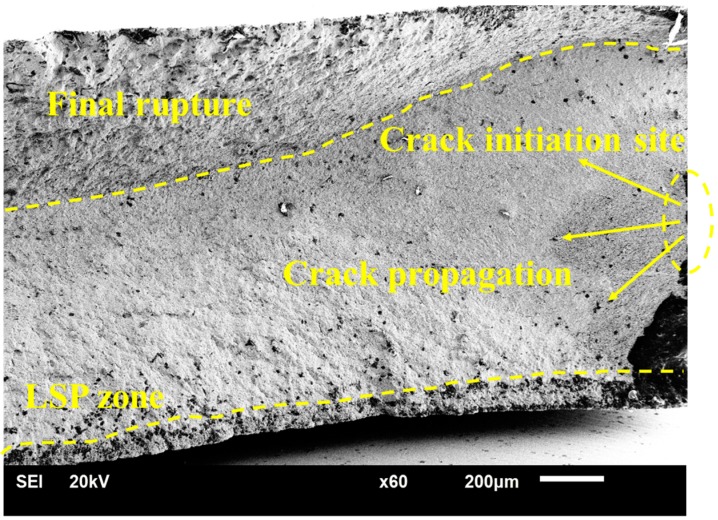
SEM diagram of fracture surface of the LSP-ed specimens.

The high-cycle fatigue fractures before and after LSP were morphologically analyzed to clarify the micro-influence mechanism of LSP on the high-cycle fatigue life. From the Figure 6, we can clearly see that the crack initiation zone is comparative smooth and the final rupture is more tortuous. The affected layer induced by LSP was marked by yellow line as shown in Figure 6. As we know, during the fatigue test, the fatigue crack usually initiates on the surface of the specimen because the decrease of grain boundary restriction and stress concentration induced by hillock or small round convex particles on the surface, then progressively expanded until final fracture [11,18,21], but the specimen treated by LSP displays different fracture features compared with the untreated specimen. Figure 6 shows the initiative place of cracks of specimens with LSP treatment. We can clearly see that the crack initiation after LSP was away from the LSP zone because of the existence of a compressive residual stress layer. This phenomenon can be explained that under the effects of compressive residual stress induced by LSP, the actual stress on the surface is well below the crack initiation stress of the specimen, and the crack nucleation is retarded. Therefore, the crack initiation sites of the samples move to the sub-surface, where actual stress is equal to the crack initiation stress. However, due to the existence of more deformation restrictions at the sub-surface, it is also difficult for fatigue cracks initiating here. And this leads to the enhancement of the fatigue crack initiation life for specimens treated by LSP.

**Figure 7:**
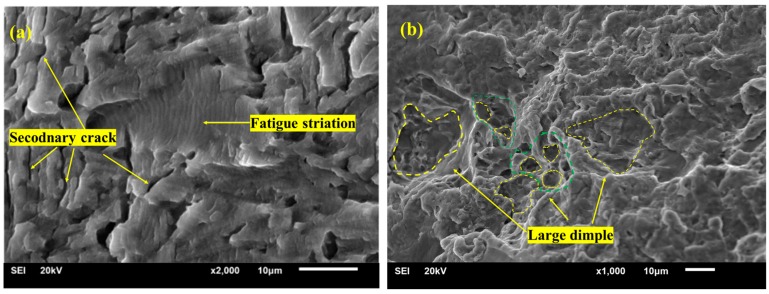
View of crack propagation region and final rupture region of Ti6Al4V specimen treated with LSP. (**a**) Crack propagation region; (**b**) Final rupture region.

The fracture morphologies in the crack expansion area and final rupture region of the LSP-ed specimens are shown in Figure 7. From Figure 7a, we can see that the high density of fatigue striations and many secondary cracks appeared on the fracture surface of the LSP-ed specimen. From some literatures we know that high density of fatigue striations indicates a lower crack propagation rate [36,37]. Besides, the secondary cracks can also help to decrease the initial microcrack growth rates because it can consume lots of energy [38]. About these viewpoints, further investigations are needed. Figure 7b shows the dimples on the final rupture region. From Figure 7b, we can see that many dimples appeared of the LSP-ed specimen. Beyond that, we can see the dimples in the final fracture area of LSP-ed specimen are more in quantity and larger in size. The larger dimple with many small dimples inserted into its inside is also observed and distributed uniformly and regularly, which suggests better toughness of Ti6Al4V titanium alloy after LSP.

## 4. Conclusions

In this paper, the effects of residual stress induced by LSP without coating on S–N curves and fracture morphology of Ti6Al4V titanium alloy were studied. The following conclusions are drawn:

1. There is a tensile residual stress appearing on the surface of the untreated specimen. After LSP, the surface residual stress is changed from tension stress to compressive stress. Besides, a deeper compressive residual stress layer is also introduced into the Ti6Al4V titanium alloy specimen.
2. The fatigue limits of the untreated and LSP-ed titanium alloys are 264 MPa and 305 MPa respectively. The fatigue limit of the specimens treated by LSP was improved by 16%. After LSP, the S–N curve shifts upward.
3. Due to the high-value compressive residual stress induced by LSP, the tensile stress produced by external loading is decreased. It is beneficial for the improvement of the crack initiation life. After LSP, the crack initiation sites of the samples moved to the subsurface. All in all, the LSP process can produce higher-amplitude compressive residual stress into the materials, which is an important factor in the improvement of the fatigue life of metallic components.

## Figures and Tables

**Table 1:** Chemical composition of Ti6Al4V titanium alloy (%) (Data from [23]).

Alloying Elements			Impurities Not Greater than						
Al	V	Ti	Fe	C	N	H	O	Other Elements	
								Individual	Sum
5.5~6.8	3.5~4.5	3.5~4.5	1.6~2.4	1.6~2.4	0.05	0.0125	0.13	0.1	0.4

**Table 2:** Physical parameters of Ti6Al4V titanium alloy (Data from [23]).

Material	Density/g·cm^−3^	Poisson’s Ratio	Elastic Model/GPa	Shear Modulus/GPa
Ti6Al4V	4.44	0.34	10^9^	44

**Table 3:** Heat treatment mechanism and basic mechanical properties of Ti6Al4V titanium alloy (Data from [23]).

Technical Standard	Heat Treatment Mechanism	$\sigma_{b}/\mathrm{MPa}$	$\sigma_{0.2}/\mathrm{MPa}$	$\delta_{5}/\%$
GJB 494-1988	Annealing: 700~850 °C, 0.5~2 h, air cooling	925	870	12

**Table 4:** LSP parameters utilized on Ti6Al4V titanium alloy.

Condition	Wavelength (nm)	Spot Size (mm)	Overlap	Pulse Energy (J)
LSP	1064	2.4	50%	4

**Table 5:** XRD parameters during residual stress measurement.

Item	Description
Radiation	Cu-Kα
Aperture size(diameter)	2 mm
Crystal plane	{213}
2θ	142°
